# Tunable optical and magneto-optical Faraday and Kerr rotations in a dielectric slab doped with double-V type atoms

**DOI:** 10.1038/s41598-020-65505-z

**Published:** 2020-05-22

**Authors:** Azar Vafafard, Mostafa Sahrai

**Affiliations:** Faculty of Physics, University of Tabriz, Tabriz, Iran

**Keywords:** Magneto-optics, Quantum optics, Photonic crystals

## Abstract

We theoretically investigate the optical and magneto-optical Faraday and Kerr rotations of a probe field that propagates through a nonmagnetic dielectric slab doped with double-V type atoms. Both rotations and corresponding ellipticities, as well as the intensities of transmitted and reflected beams, are modified by quantum coherence induced in the atomic system. We show that applying a control laser field makes the system optically active and simultaneously, transparent to one component of the probe field. We demonstrate that the response of the slab can be modified both electrically and magnetically. Applying the second control laser field with different Rabi frequencies improves the optical properties of the slab due to the induced coherent effects. We present analytical expressions for facilitating the detailed study of the system behaviors. Magneto-optical Faraday rotation 45° with transmission close to $100 \%$ and large Kerr rotation with high reflection are significant results from the influence of both the control and magnetic fields on such a small structure. By prevailing over the tradeoff between reflection and rotation, the proposed model could be considered as a special candidate for rotating the polarization plane of the transmitted and reflected beams, simultaneously.

## Introduction

Light polarization rotation is well known as an optical process resulting from the passage of light beam through a chiral medium. This phenomenon is called optical activity when chirality is due to the helicity of the molecule’s structure^1^. Applying a magnetic field can also make chirality caused by the Zeeman interaction in an initially isotropic medium. Physically, Zeeman splitting of m-degenerate magnetic sub-levels leads to an asymmetry in susceptibility of the medium for two left and right-handed circular polarization components of light. Consequently, polarization rotation is implemented by the magnetic field induced birefringence or dichroism. Such rotation of light polarization is called magneto-optical rotation (MOR)^2^. MOR has a key role in varied areas such as control of light polarization, Faraday optical isolators^3–5^, magnetometry^6^, and laser frequency stabilization^7^.

Laser-driven isotropic atomic vapor is also known to exhibit polarization rotation that is called optical Faraday rotation (OFR)^1^. Laser-induced chirality originates from a Zeeman-like AC Stark shift. In this case, the polarization rotation depends on the control field parameters. Gaseous systems have been the subject of extensive both theoretically and experimentally OFR studies by reason of the strong resonance and hence large OFR in such systems. In 1976, Liao *et al*.^8^ first showed the rotation of polarization due to the two-photon resonance process in sodium. Significant atomic birefringence and electromagnetically induced transparency were demonstrated in a ladder-type of metastable helium atoms^9^. In an interesting experiment, the laser-induced anisotropy and OFR were observed in a cascade configuration of ytterbium atoms^10^. It has been also shown the enhancement of MOR of polarized light by both the numerical and analytical calculations. Patnaik and Agarwal theoretically studied the effect of both control laser fields and a static magnetic field on the polarization rotation^11,12^. More recently, OFR rotation was studied in a closed-loop atomic system that provides facilities for controlling the optical properties by the relative phase of applied laser fields^13^.

On the other hand, it is well-known that the polarization plane of the reflected light from a magnetic medium is also experienced rotation. This effect, so-called magneto-optical Kerr effect (MOKE), has attracted attention in the fabrication of magneto-optical data storage disks and material characterization. It is worth noting that the MOKE is very small in many magnetic media due to the limited lengths of the interaction region. Enhancement of such an effect would provide interesting applications to applied physics. Examples include mirrors with light rotation ability and circulators in nonreciprocal devices. The observations show that increment of rotation angles is accompanied by a decrease in transmitted and reflected light. Many efforts have been appropriated to enhance both optical Faraday^14,15^ and Kerr rotations^16,17^ with large reflection and transmission. Recently, the study of optical MOR and MOKE in dielectric slabs and photonic crystals including magnetic media has attracted much attention to achieve the enhanced rotation angle with high transmission. In fact, the propagation of electromagnetic waves through a photonic crystal has been investigated for many potential applications due to the resonant effects of periodic structures. Moreover, the properties of photonic crystals allow developing much smaller and cheaper circulator than bulk devices^18–20^. It has been shown that designing a repeated identical structure with defect layers leads to the improvement of transmission with enhanced rotation. One of the most exciting results was obtained in a theoretical study in which the arbitrary Kerr rotations are achieved with high reflection in a photonic crystal^21^.

In the present study, we propose a novel scheme to enhance the optical Faraday and Kerr rotations, simultaneously. The idea is to dope double-V type atoms into the slab for inducing anisotropy via either control laser fields or a magnetic field. Modification of the atomic structure by the control laser fields provides the possibility of generation and manipulation of polarization rotation in atoms and consequently in the nonmagnetic slab even in the absence of a magnetic field. In a conventional gas system, only rotation of the polarized light passing through the atomic system is obtained, whereas in a dielectric slab or photonic crystals the rotation of reflected light is also achieved. Furthermore, strong resonance in atomic systems leads to strong OFR and optical Kerr effect (OKE). Optimization of OKE and MOKE via induced quantum coherence in doped atoms is a fascinating advantage of our proposed system. We find that using a circularly polarized control field can make the medium transparent to only one of the probe field polarization components and therefore the nonmagnetic slab turns to an optically active and transparent medium for light propagation. Applying the second control field to the atomic system leads to the scattering of both control fields in the probe frequency and cross-Kerr effect that enhance the OFR and Kerr rotations. We also examine the transmission, reflection and corresponding polarization rotations and ellipticities in the presence of a magnetic field. By the addition of the magnetic field to the system, MOF ${45}^{\circ }$ with transmission close to $100 \%$ and large MOKE with high reflection as the great results are achieved.

## Summary of mathematical model

### Linearly polarized light propagation in a dielectric slab

**Figure 1:**
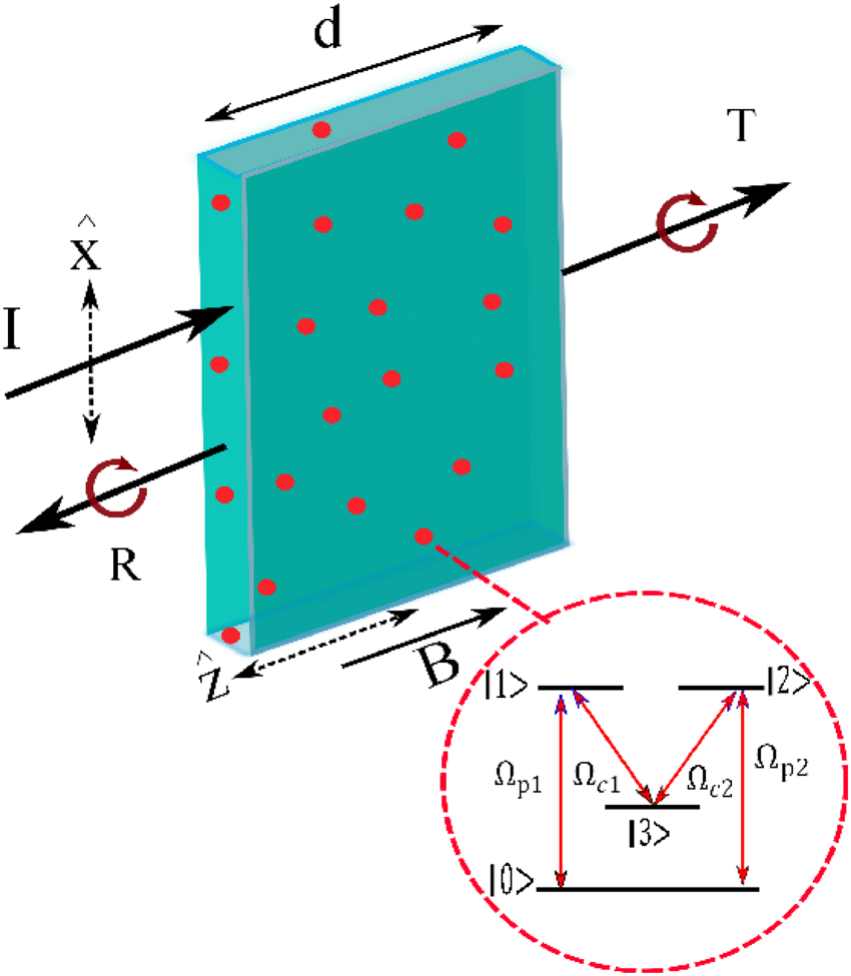
Schematic diagram of the slab doped with double-V type atoms. The magnetic field $\overrightarrow{{B}_{m}}$ is in *z* direction and the input field is x-polarized.

We consider the propagation of a linearly polarized probe field ${\overrightarrow{E}}_{p}$ with frequency ${\omega }_{p}$, through a nonmagnetic dielectric slab as shown in Fig. 1. The probe field, that propagates along the axis $z$, is written as

1 $${\overrightarrow{E}}_{p}(z,t)=\{A(z)\,{e}^{-i({\omega }_{p}t-{k}_{p}z)}+B(z)\,{e}^{i({\omega }_{p}t-{k}_{p}z)}\}+c.\,c,$$

where

2 $$\overrightarrow{A}(z)={A}_{x}(z)\,\hat{x}+{A}_{y}(z)\,\hat{y},$$

3 $$\overrightarrow{B}(z)={B}_{x}(z)\,\hat{x}+{B}_{y}(z)\,\hat{y}$$

are the amplitude of the forward and backward waves, respectively. The slab is extended from $z=0$ to $z=d$ and is placed in the vacuum. In the proposed model, the slab is doped by four-level atoms that are the source of birefringence and dichroism in the medium. Calculation of the transmission ${T}_{\pm }$ and reflection ${R}_{\pm }$ coefficients is the aim of this section. For the best expression of the problem, the propagated field amplitudes are resolved into two circularly polarized ${\sigma }_{+}$ and ${\sigma }_{-}$ components

4 $${C}_{\pm }=({A}_{x}\pm i\,{A}_{y})/\sqrt{2},$$

5 $${D}_{\pm }=({B}_{x}\pm i\,{B}_{y})/\sqrt{2}.$$

Here, the boundary conditions ${A}_{x}(0)=1$ and ${A}_{y}(0)={B}_{x}(d)={B}_{y}(d)=0$ are assumed in order to represent a TE wav as the incident field on the slab. Using Eqs. 4 and 5, the circularly polarized amplitudes of incident filed are given by ${C}_{+}(0)={C}_{-}(0)=1/\sqrt{2}$.

The dielectric function of the considered slab consists of two parts, the background dielectric constant and the susceptibility of doped atoms. If the electric susceptibilities of doped atoms for the two circularly polarized components of the probe field are shown by ${\chi }_{+}$ and ${\chi }_{-}$, the dielectric functions can be expressed as

6 $${\varepsilon }_{\pm }({\omega }_{p})={\varepsilon }_{b}+{\chi }_{\pm }({\omega }_{p}),$$

where ${\varepsilon }_{b}={n}_{b}^{2}$ denotes the background refractive index of the slab. Here, the birefringence can be induced in a nonmagnetic slab because of applying the laser and magnetic fields to the atoms.

We study the propagation of the light beam through the slab based on the transfer matrix formulation. It is shown that for a TE wave, any two positions $z$ and $z+\delta z$ in a slab are related by the transfer matrix

7 $$M(\delta z,\omega )=\left(\begin{array}{cc}\cos (k\,\delta z) & i\,\frac{1}{n({\omega }_{p})}\,\sin (k\,\delta z)\\ i\,n({\omega }_{p})\,\sin (k\,\delta z) & \cos (k\,\delta z)\end{array}\right),$$

where $k$ is the wave vector along the $z$ axis and $n(\omega )=\sqrt{\varepsilon }$ shows the refractive index of the slab. Using the transfer matrix method, the transmission and reflection coefficients for right and left circular polarized components of the probe field are identified as

8 $${t}_{\pm }=\frac{1}{\cos (k\,d)-\frac{i}{2}(\frac{1}{\sqrt{{\varepsilon }_{\pm }}}+\sqrt{{\varepsilon }_{\pm }})\,\sin (k\,d)},$$

9 $${r}_{\pm }=\frac{-\frac{i}{2}(\frac{1}{\sqrt{{\varepsilon }_{\pm }}}-\sqrt{{\varepsilon }_{\pm }})\sin (k\,d)}{\cos (k\,d)-\frac{i}{2}(\frac{1}{\sqrt{{\varepsilon }_{\pm }}}+\sqrt{{\varepsilon }_{\pm }})\,\sin (k\,d)}.$$

It is obviously seen that the reflection and transmission depend on the thickness and refractive index of the slab. In this study, we consider the resonance condition in which the thickness of the slab is assumed as $d=2m(\lambda /4\sqrt{{\varepsilon }_{b}})$. This thickness is an even number ($2m$) of quarter-wave plates of length $(\lambda /4\sqrt{{\varepsilon }_{b}})$. Here, $\lambda$ is the wavelength of the probe field.

After solving the problem for individual circular components, the transmission and reflection coefficients in terms of linear polarization can be calculated via combination of Eqs. 4 and 5 with following equations^18^:

10 $${t}_{\pm }=|{t}_{\pm }|{e}^{i{\phi }_{\pm }}=\frac{{C}_{\pm }(d)}{{C}_{\pm }(0)},$$

11 $${r}_{\pm }=|{r}_{\pm }|{e}^{i{\psi }_{\pm }}=\frac{{D}_{\pm }(0)}{{C}_{\pm }(0)}.$$

Then, the total transmission $T$ and reflection $R$ coefficients are written as

12 $$T={|{A}_{x}(d)|}^{2}+{|{A}_{y}(d)|}^{2},$$

13 $$R={|{B}_{x}(0)|}^{2}+{|{B}_{y}(0)|}^{2},$$

From the obtained coefficients, the Faraday (F) and Kerr (K) rotations and corresponding ellipticities are given as^18^

14 $${\theta }_{F}=\frac{1}{2}{\tan }^{-1}\frac{2\,\mathrm{Re}[\frac{{A}_{y}(d)}{{A}_{x}(d)}]}{1-{|\frac{{A}_{y}(d)}{{A}_{x}(d)}|}^{2}},\,{\eta }_{F}=\,\tan \left[\frac{1}{2}{\sin }^{-1}\frac{-2\,\text{Im}\,[\frac{{A}_{y}(d)}{{A}_{x}(d)}]}{1+{|\frac{{A}_{y}(d)}{{A}_{x}(d)}|}^{2}}\right],$$

15 $${\theta }_{K}=\frac{1}{2}{\tan }^{-1}\frac{2\,\mathrm{Re}[\frac{{B}_{y}(0)}{{B}_{x}(0)}]}{1-{|\frac{{B}_{y}(0)}{{B}_{x}(0)}|}^{2}},\,{\eta }_{K}=\,\tan \left[\frac{1}{2}{\sin }^{-1}\frac{-2\,\text{Im}\,[\frac{{B}_{y}(0)}{{B}_{x}(0)}]}{1+{|\frac{{B}_{y}(0)}{{B}_{x}(0)}|}^{2}}\right].$$

### Susceptibilities of the atoms

Now, we present the detailed description of the four-level double-V type atomic system as depicted in Fig. 1. The system includes two ground states $|0\rangle$ and $|3\rangle$ and two degenerate excited states $|1\rangle$ and $|2\rangle$. Four dipole-allowed transitions, $|0\rangle -|1\rangle$, $|0\rangle -|2\rangle$, $|3\rangle -|1\rangle$ and $|3\rangle -|2\rangle$ are considered to make a closed-loop scheme in this study. The transition $|1\rangle -|0\rangle$ ($|2\rangle -|0\rangle$) is coupled by the left (right) circular component of the linear polarized probe field. The Rabi frequency of the probe field is ${\Omega }_{p1}={\overrightarrow{E}}_{p}.{\mu }_{01}/\hslash$(${\Omega }_{p2}={\overrightarrow{E}}_{p}.{\mu }_{02}/\hslash$). Also, a control field ${E}_{c1}={\overrightarrow{E}}_{c1}{e}^{-i({\omega }_{c1}t-{k}_{c1}z-{\phi }_{c1})}+c.\,c$(${E}_{c2}={\overrightarrow{E}}_{c2}{e}^{-i({\omega }_{c2}t-{k}_{c2}z-{\phi }_{c2})}+c.\,c$) with Rabi frequency ${\Omega }_{c1}={\overrightarrow{E}}_{c1}.{\mu }_{31}/\hslash$ (${\Omega }_{c2}={\overrightarrow{E}}_{c2}.{\mu }_{32}/\hslash$) is applied to the transition $|1\rangle -|3\rangle$ ($|2\rangle -|3\rangle$) that requires a left (right)-handed circularly polarized field for excitation. The parameter ${\mu }_{ij}=\langle i|-e\,{\bf{r}}|j\rangle$ denotes the dipole matrix element of $|i\rangle -|j\rangle$ transition. Such a system can be generated in ${D}_{1}$ transitions of ${}^{87}Rb$ atoms. Lower levels are two hyperfine levels $|0\rangle =5{S}_{1/2}(F=1,\,{m}_{F}=0)$ and $|3\rangle =5{S}_{1/2}(F=2,\,{m}_{F}=0)$ separated by $\,6.83\,GHz$. The levels $|1\rangle =5{P}_{1/2}(F{\prime} =2,\,{m}_{F}=-\,1)$ and $|2\rangle =5{P}_{1/2}(F{\prime} =2,\,{m}_{F}=1)$ are chosen as the upper levels^22,13^. $F$ and $F{\prime}$ are the total atomic angular momentum quantum numbers and ${m}_{F}$ is the magnetic quantum number of corresponding state.

The Hamiltonian of the atom interacting with the probe and control fields in the rotating wave and dipole approximations can be written as

16 $${{\rm H}}_{I}=-\,[{\Omega }_{p1}{e}^{-i{\delta }_{p}t}|1\rangle \langle 0|+{\Omega }_{p2}{e}^{-i{\delta }_{p}t}|2\rangle \langle 0|+{\Omega }_{c1}{e}^{-i{\delta }_{c1}t}|1\rangle \langle 3|+{\Omega }_{c2}{e}^{-i{\delta }_{c2}t}|2\rangle \langle 3|]+H.c.\,,$$

where ${\delta }_{p1}={\omega }_{p}-{\omega }_{10},\,{\delta }_{p2}={\omega }_{p}-{\omega }_{20}$, ${\delta }_{c1}={\omega }_{c1}-{\omega }_{13}$, ${\delta }_{c2}={\omega }_{c2}-{\omega }_{23}$. Here ${\omega }_{p}$ is the frequency of the probe field, ${\omega }_{c1}$ and ${\omega }_{c2}$ are frequencies of the control fields, and the parameter ${\omega }_{ij}$ shows the central frequency of $|i\rangle -|j\rangle$ transition.

Using the von Neumann equation $\partial \rho /\partial t=-\frac{i}{\hslash }[{H}_{in},\rho ]-\frac{1}{2}\{\varGamma ,\rho \}$, the equations of motion in the rotating-wave approximation and rotating frame can be derived as

$${\dot{\rho }}_{00}={\gamma }_{10}{\rho }_{11}+{\gamma }_{20}{\rho }_{22}+i\,{\Omega }_{p1}^{\ast }{\rho }_{10}-i\,{\Omega }_{p1}{\rho }_{01}+i\,{\Omega }_{p2}^{\ast }{\rho }_{20}-i\,{\Omega }_{p2}{\rho }_{02},$$

$${\dot{\rho }}_{11}=-({\gamma }_{10}+{\gamma }_{13}){\rho }_{11}+i{\Omega }_{p1}{\rho }_{01}-i\,{\Omega }_{p1}^{\ast }{\rho }_{10}-i\,{\Omega }_{c1}^{\ast }{\rho }_{13}+i\,{\Omega }_{c1}{\rho }_{31},$$

$${\dot{\rho }}_{22}=-({\gamma }_{20}+{\gamma }_{23}){\rho }_{22}+i{\Omega }_{p2}{\rho }_{02}-i\,{\Omega }_{p2}^{\ast }{\rho }_{20}-i\,{\Omega }_{c2}^{\ast }{\rho }_{23}+i\,{\Omega }_{c2}{\rho }_{32},$$

$${\dot{\rho }}_{10}=(-{\varGamma }_{10}+i\,{\delta }_{p}){\rho }_{10}+i\,{\Omega }_{p1}({\rho }_{00}-{\rho }_{11})-i\,{\Omega }_{p2}{\rho }_{12}-i\,{\Omega }_{c1}{\rho }_{30},$$

$${\dot{\rho }}_{12}=-\,{\varGamma }_{12}{\rho }_{12}+i\,{\Omega }_{p1}{\rho }_{02}-i\,{\Omega }_{p2}^{\ast }{\rho }_{10}+i\,{\Omega }_{c1}{\rho }_{32}-i\,{\Omega }_{c2}^{\ast }{\rho }_{13},$$

$${\dot{\rho }}_{20}=(-{\varGamma }_{20}+i\,{\delta }_{p}){\rho }_{20}+i\,{\Omega }_{p2}({\rho }_{00}-{\rho }_{22})-i\,{\Omega }_{p1}{\rho }_{21}+i\,{\Omega }_{c2}{\rho }_{30},$$

$${\dot{\rho }}_{23}=(-{\varGamma }_{23}+i\,{\delta }_{c2}){\rho }_{23}+i\,{\Omega }_{p2}{\rho }_{03}-i\,{\Omega }_{c1}{\rho }_{21}-i\,{\Omega }_{c2}({\rho }_{22}-{\rho }_{33}),$$

$${\dot{\rho }}_{13}=(-{\varGamma }_{13}+i\,{\delta }_{c1}){\rho }_{13}+i\,{\Omega }_{p1}{\rho }_{03}-i\,{\Omega }_{c1}({\rho }_{11}-{\rho }_{33})\,-i\,{\Omega }_{c2}{\rho }_{12},$$

$${\dot{\rho }}_{30}=i\,({\delta }_{p}-{\delta }_{c})-{\varGamma }_{30}{\rho }_{30}+i\,{\Omega }_{c1}^{\ast }{\rho }_{10}-i\,{\Omega }_{p1}{\rho }_{31}+i\,{\Omega }_{c2}^{\ast }{\rho }_{20}-i\,{\Omega }_{p2}{\rho }_{32},$$

17 $${\rho }_{00}+{\rho }_{11}+{\rho }_{22}+{\rho }_{33}=1\,,$$

where ${\gamma }_{ij}$ is decay rate from level $|i\rangle$ to $|j\rangle$, and ${\varGamma }_{ij}$ is the dephasing rate between levels $|i\rangle$ and $|j\rangle$. Here, the multiphoton resonance condition $({\delta }_{p1}-{\delta }_{c1}-{\delta }_{p2}+{\delta }_{c2}=0)$ is fulfilled.

The probe light with linear polarization decomposes into two opposite circular polarization components and couple the corresponding transitions. Due to the control laser field induced birefringence, the response of the atomic system to the probe laser components will be different. The atomic susceptibilities corresponding to the right and left circular polarization, that determined the response of the system, are given by

18 $${\chi }_{+}=(\frac{2{\alpha }_{0}{\gamma }_{20}}{{k}_{p}{\Omega }_{p2}})\,{\rho }_{20},$$

19 $${\chi }_{-}=(\frac{2{\alpha }_{0}{\gamma }_{10}}{{k}_{p}{\Omega }_{p1}})\,{\rho }_{10},$$

where ${\alpha }_{0}=N\,{D}^{2}{\omega }_{p}/(2\,c\hslash {\varepsilon }_{0}{\gamma }_{10})$. $N$ is the atomic number density. In the following numerical calculations, we assume $\frac{2{\alpha }_{0}{\gamma }_{j0}}{{k}_{p}{\Omega }_{pj}}\cong 1,\,j=1,2$.

In the proposed model, the role of an external magnetic field in the rotation of a probe field polarization plane can also be taken into account. By applying a magnetic field, the degeneracy of $|1\rangle$ and $|2\rangle$ is lifted. In this case, the excited energy levels differ by $2\hslash {\Delta }_{B}$ where ${\Delta }_{B}=g\mu {B}_{m}$. Here, $g$ is the Lande g factor, $\mu$ indicates the Bohr magneton and ${B}_{m}$ is the magnetic field. In the presence of the external magnetic field, the detuning of the applied fields from the corresponding atomic transitions are given by ${\delta }_{c1}=({\omega }_{c1}-{\omega }_{13}-{\varDelta }_{B})$, ${\delta }_{c2}=({\omega }_{c2}-{\omega }_{23}-{\Delta }_{B})$, ${\delta }_{p1}=({\omega }_{p}-{\omega }_{10}-{\Delta }_{B})$, and ${\delta }_{p2}=({\omega }_{p}-{\omega }_{20}+{\Delta }_{B})$.

For precisely physical description, we solve the density matrix equations to obtain the probe susceptibilities under the weak probe field approximation as

20 $${\rho }_{20}=\frac{2\,{\Omega }_{p}[(i\,\gamma +2{\delta }_{p})({\delta }_{p}-{\delta }_{c})+2({\delta }_{p}-{\delta }_{c}){\Delta }_{B}+2{\Omega }_{c1}^{\ast }({\Omega }_{c2}-{\Omega }_{c1})]}{A},$$

21 $${\rho }_{10}=\frac{2\,{\Omega }_{p}[(i\,\gamma +2{\delta }_{p})({\delta }_{p}-{\delta }_{c})-2({\delta }_{p}-{\delta }_{c}){\Delta }_{B}+2{\Omega }_{c2}^{\ast }({\Omega }_{c1}-{\Omega }_{c2})]}{A},$$

where $A=4({\delta }_{p}-{\delta }_{c})\,{\Delta }_{B}^{2}+(\gamma -2\,i\,{\delta }_{p})[(\gamma -2i{\delta }_{p})({\delta }_{p}-{\delta }_{c})+2i{|{\Omega }_{c1}|}^{2}+2i{|{\Omega }_{c2}|}^{2}]+4{\Delta }_{B}({|{\Omega }_{c2}|}^{2}-{|{\Omega }_{c1}|}^{2})$. These relation are calculated under the condition, ${\omega }_{c2}-{\omega }_{23}={\omega }_{c1}-{\omega }_{13}={\delta }_{c}$, ${\omega }_{p}-{\omega }_{10}={\omega }_{p}-{\omega }_{20}={\delta }_{p}$, ${\Omega }_{p1}={\Omega }_{p2}={\Omega }_{p}$, ${\gamma }_{20}={\gamma }_{10}=\gamma /4$ and ${\gamma }_{23}={\gamma }_{13}=3\gamma /4$. In Eq. 20, the first component of three contributions shows a direct scattering of the probe field. The second term exhibits the effect of the magnetic field on the susceptibilities. The first part of the last term proportional to ${\Omega }_{c1}^{\ast }{\Omega }_{c2}$ represents the scattering of both control fields into the probe field frequency. Moreover, the second part of the third term demonstrates the cross-Kerr effect of the second control field in the probe mode. The similar analytical expression admits the same physical interpretation for ${\rho }_{10}$.

## Results and Discussion

We now turn to the numerical study of the reflection, transmission, polarization rotations and ellipticities of the forward and backward lights in the proposed dielectric slab. First, we focus on the properties of the slab in the absence of the magnetic field. In this case, the polarization plane rotates because of the laser field induced asymmetry. The effects of the probe field detuning and Rabi frequency of the control field on the response of the slab to the probe field are investigated. The center frequency of the probe field is ${\omega }_{p}={\omega }_{10}=2\pi \times 377{\rm{.107THz}}$, the background dielectric is ${\varepsilon }_{b}=4$, and the decay rate of ${D}_{1}$ transitions in ${}^{87}Rb$ atoms is $\gamma =2\pi \times 5{\rm{.746MHz}}$. All parameters are scaled in units of $\gamma$.

**Figure 2:**
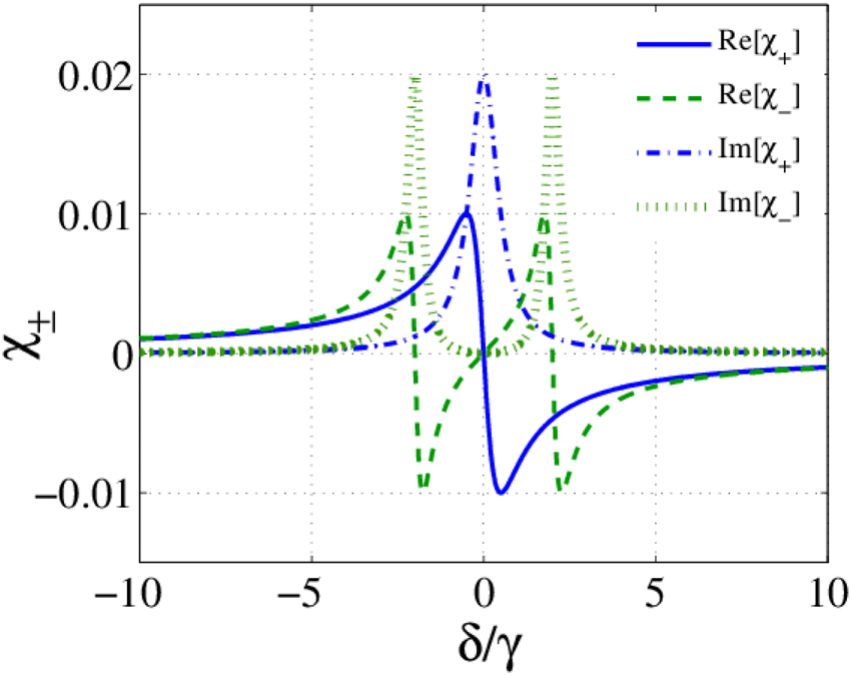
Imaginary and real parts of ${\chi }_{\pm }$ versus the probe field detuning. The used parameters are ${\gamma }_{20}={\gamma }_{10}=\gamma /4$, ${\gamma }_{23}={\gamma }_{13}=3\gamma /4$, ${\Omega }_{p1}={\Omega }_{p2}=0.01\gamma$, ${\Omega }_{c1}=2\gamma$, ${\Omega }_{c2}=0$, and ${\Delta }_{B}={\delta }_{c}=0$.

We first, set ${\Omega }_{c2}=0$ and plot the susceptibility components of atoms in Fig. 2. The atomic parameters are ${\gamma }_{20}={\gamma }_{10}=\gamma /4$, and ${\gamma }_{23}={\gamma }_{13}=3\gamma /4$. The used optical parameters are ${\Omega }_{p1}={\Omega }_{p2}=0.01\gamma$, ${\Omega }_{c1}=2\gamma$, and ${\Delta }_{B}={\delta }_{c}=0$.

**Figure 3:**
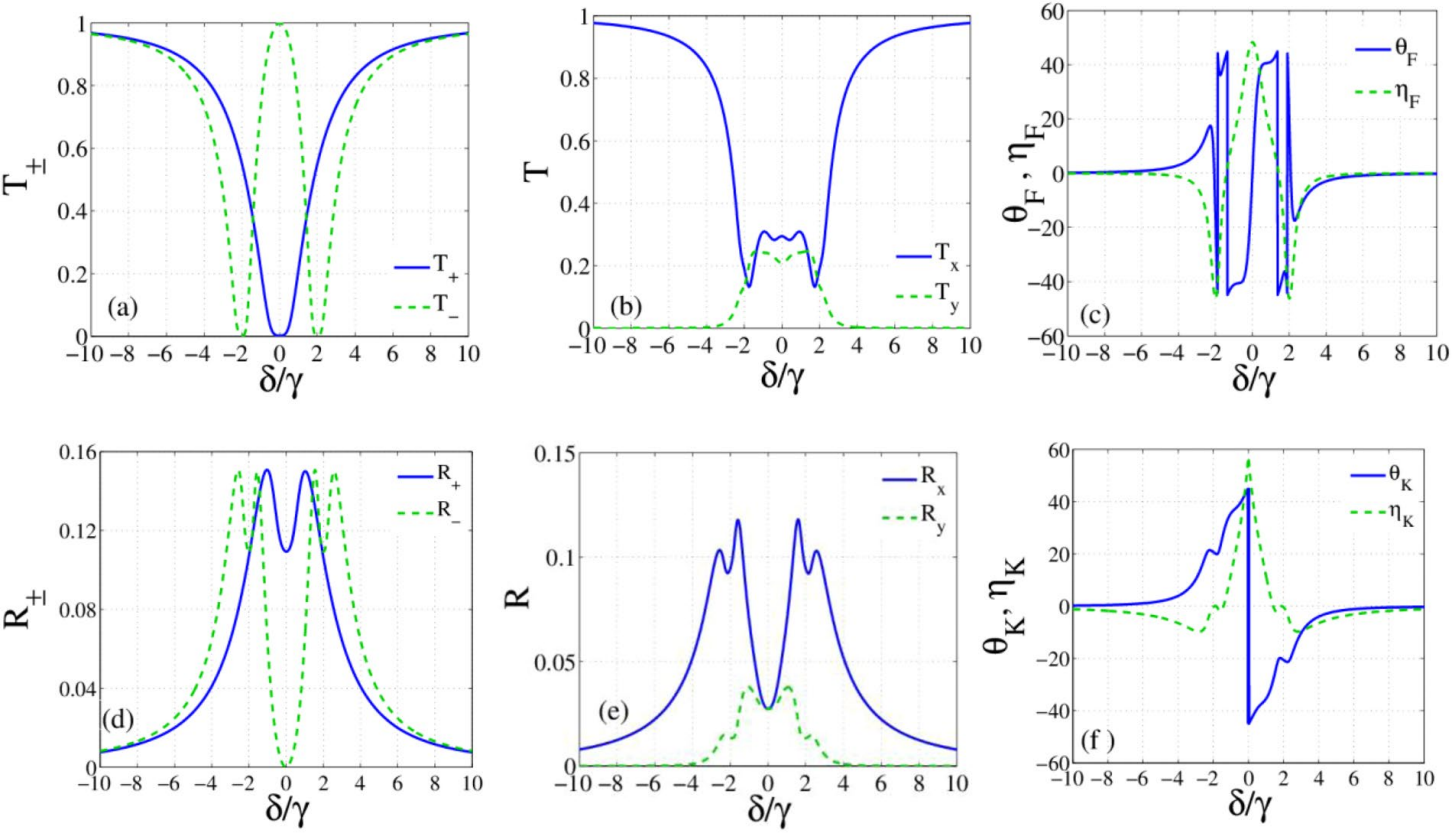
Response of the slab for resonance thickness. First and second rows show the transmitted and reflected light properties, respectively. Transmission (**a**) and reflection (**d**) of circularly polarized modes, transmission (**b**) and reflection (**e**) of linearly polarized modes, OFR and corresponding ellipticity (**c**), OKE and corresponding ellipticity (**f**) versus the detuning of the probe field. m = 400, and the parameters are the same with Fig. 2.

Figure 2 shows the establishment of Autler-Townes effect in the response of the atomic system to the left circular component of the probe field. So, the medium is transparent to one of the circularly polarized components of the probe field but is still opaque to another component. The corresponding transmission and reflection of circularly (a, c) and linearly (b, d) polarized modes of the probe field are presented in Fig. 3. As expected from the analysis of the atomic response, the transmitted energy of the right circularly polarized component (${T}_{+}$) of the probe field becomes zero at $\delta =0$ because of the strong absorption (Fig. 3(a)). While, the left circular component is completely transmitted through the transparent medium. It is clearly seen that the linearly polarized light in the resonance situation becomes elliptically polarized after transmission through the slab. Figure 3(d) shows that the reflected resonant light from the proposed slab is also an elliptically polarized light in the opposite direction. The maximum ellipticities with the zero OFR and OKE of resonant light in Fig. 3(c,f) are in conformity with these results. Also, Fig. 3(b) shows that the maximum transformation to TM mode is accomplished in $\delta =\pm 1.4\,\gamma$ where the circular transmitted energies are the same and. The peak of TM mode of the reflected light is also achieved in the same detuning, as shown in Fig. 3(e). Note that two circular modes are reflected with equal intensities and zero ellipticity in $\delta =\pm 1.4\,\gamma$. Therefore, applying just one control field can make the medium both transparent and optically active in the proposed system.

**Figure 4:**
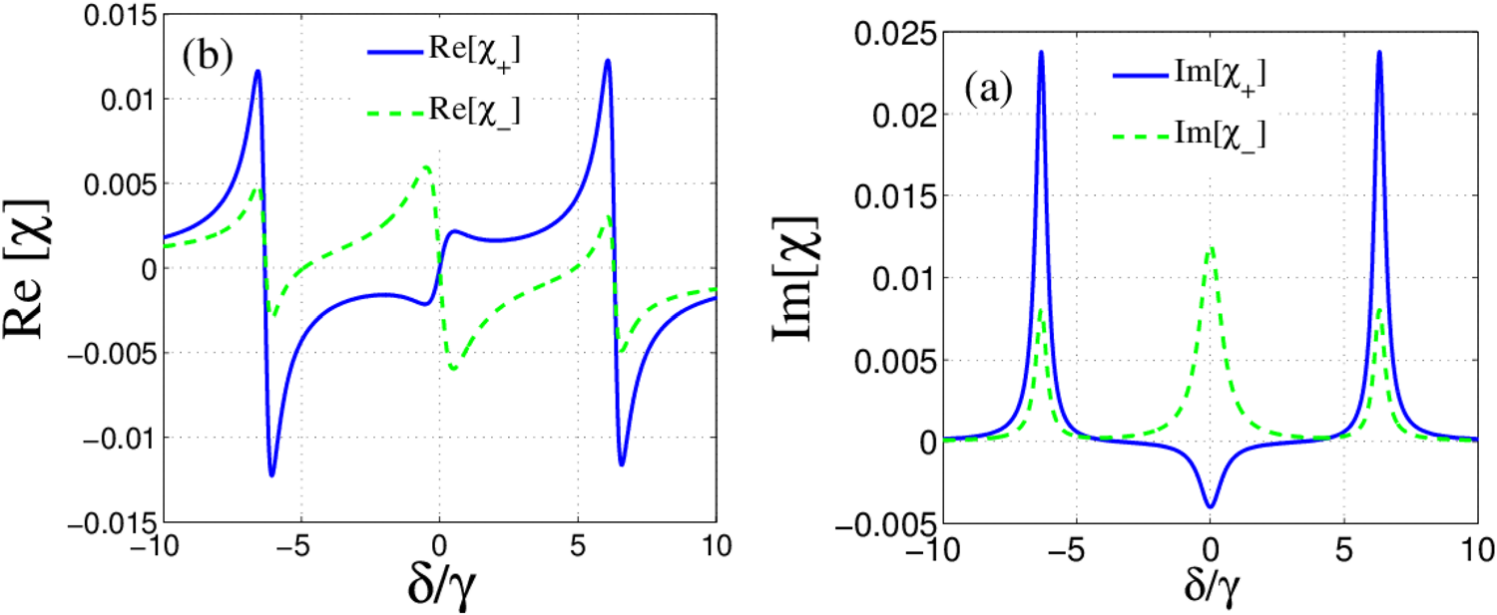
Imaginary (**a**) and real parts (**b**) of ${\chi }_{\pm }$ versus the detuning of the probe field in the absence of external magnetic field for ${\Omega }_{c1}=2\gamma$, ${\Omega }_{c2}=6\gamma$. The other parameters are the same with Fig. 2.

By switching on the second control field, we now investigate the effect of both control fields on the response of the slab. We plot the imaginary (Fig. 4 (a)) and real (Fig. 4 (b)) parts of the susceptibilities for ${\Omega }_{c2}=3\,{\Omega }_{c1}=6\gamma$. Figure 4(a) shows the amplification of the left circular component of the probe field in $\delta =0$. Note that if $\text{Im}[\chi ({\omega }_{p})] < 0$, the atomic system causes the amplification of the probe beam while for $\text{Im}[\chi ({\omega }_{p})] > 0$ the probe beam is attenuated. It is worth mentioning that the medium with gain provides the possibility of the transmission larger than unity. Moreover, an investigation on Fig. 4 reveals that the splitting of two modes around $\delta =0$ is due to dichroism while it occurs around $\delta =4\,\gamma$ because of the birefringence.

**Figure 5:**
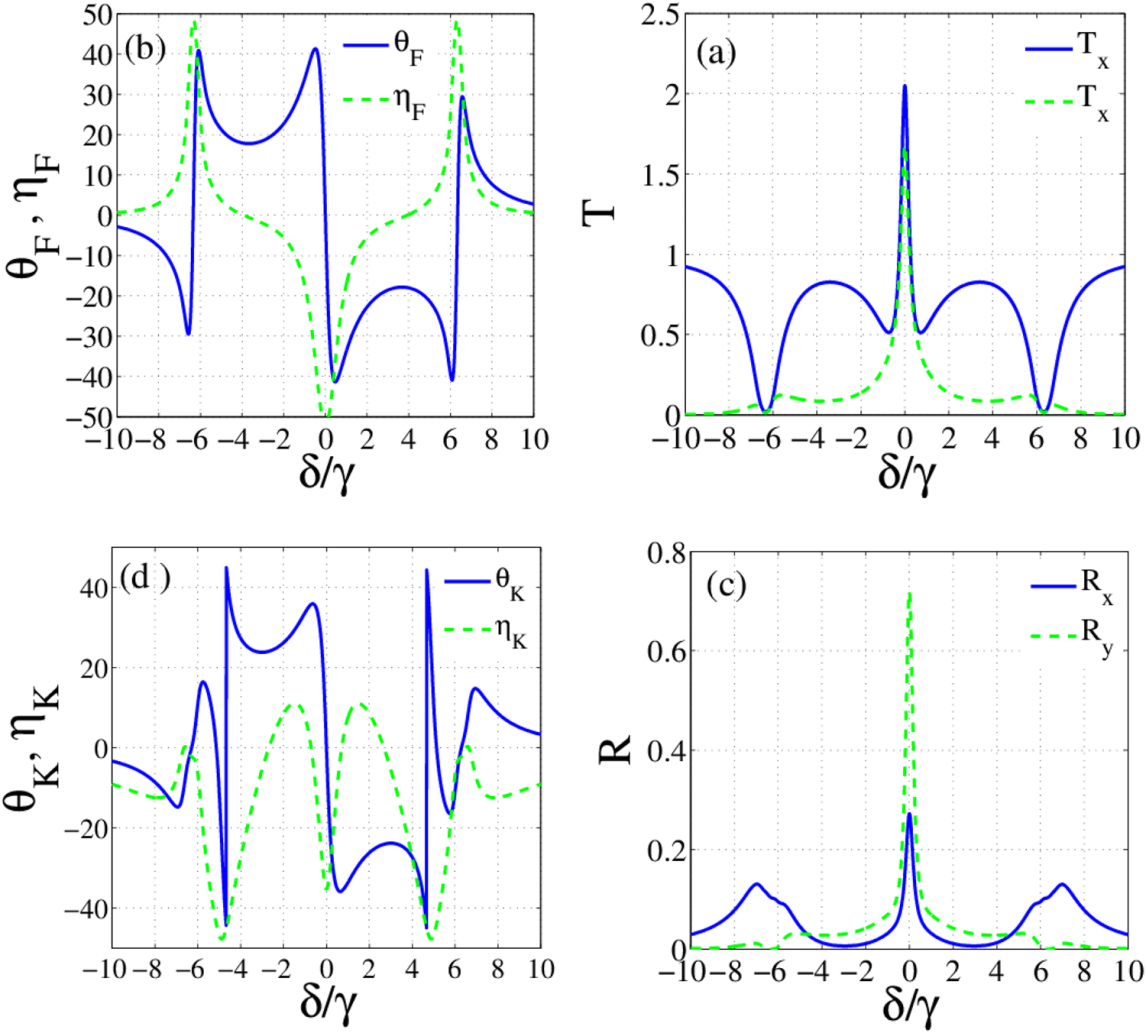
Transmission of linearly polarized modes (**a**), OFR and corresponding ellipticity (**b**) reflection of linearly polarized modes (**c**) OKE and corresponding ellipticity (**d)** versus the detuning of the probe field. The other parameters are the same with Fig. 3.

We display the transmission of linearly polarized modes in Fig. 5(a) and OFR with ellipticity in Fig. 5(b) as a function of the probe field detuning. The amplitude variations between the right and left circularly-polarized light at $\delta =0$ causes the large ellipticity. While the zero phase variation between two circularly polarized modes leads to a very small OFR. Polarization rotation $\mp {15}^{\circ }$ with transmission close to 100% is the maximum achieved rotation angle with zero ellipticity that occurs in $\delta =\pm 4\,\gamma$ for selected parameters. As illustrated in Fig. 5(c,d), the OKE $-{45}^{\circ }$ and ${45}^{\circ }$ are available here, but such rotations are accompanied with large ellipticities and small reflections. The OKEs that are accompanied with high reflections and zero ellipticities occur around $\delta =\pm 0.7\gamma$. In this detuning of the probe field, the reflection for OKE $\mp {35}^{\circ }$ is about $20$%. Physically, the closed laser-field interaction loop provides the scattering of both control fields into the probe field mode. Scattering of both control fields in the probe mode leads to the enhancement of intensities of reflected and transmitted light beams in the vicinity of the resonance.

**Figure 6:**
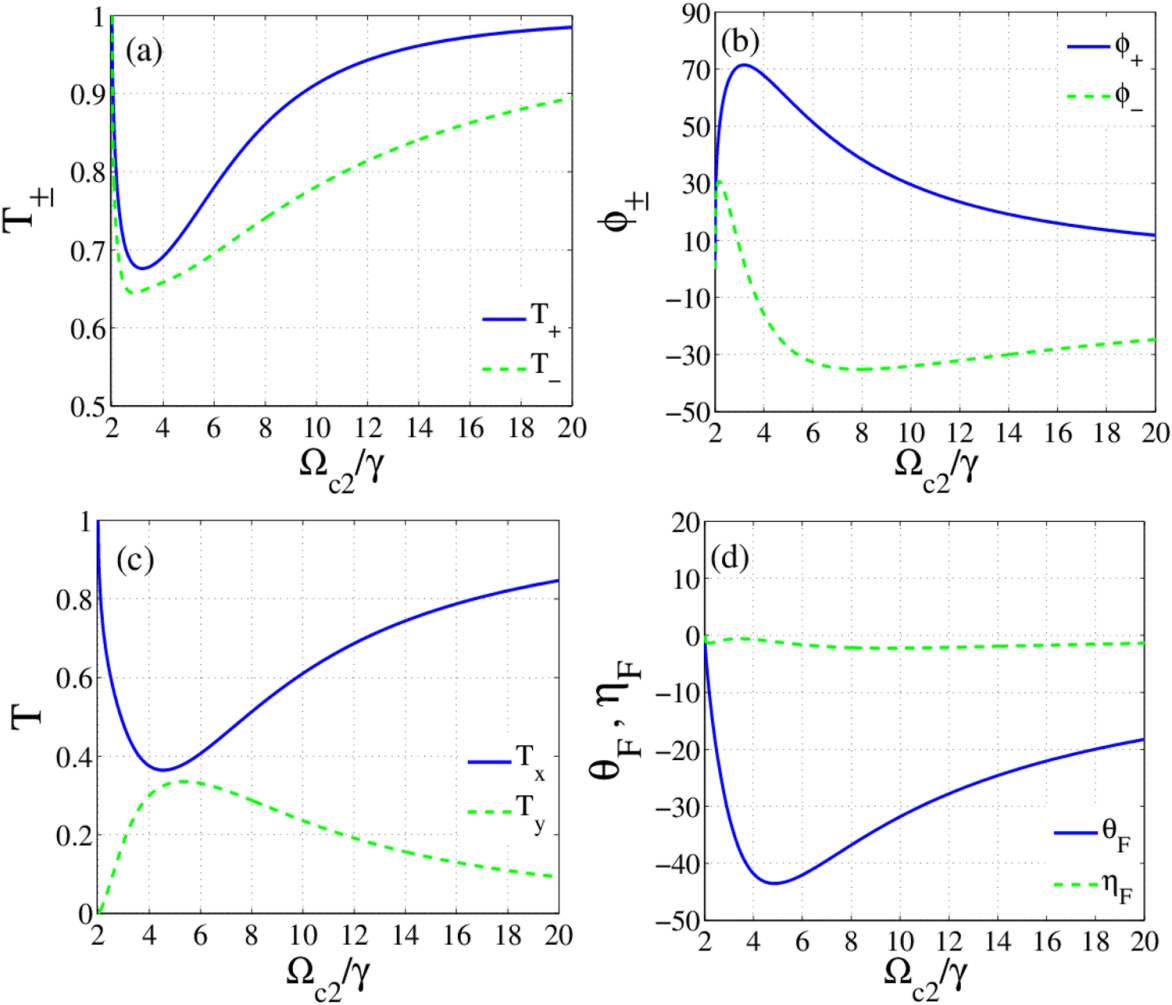
Transmission of circularly polarized modes (**a**), phase profiles (**b**), transmission of linearly polarized modes (**c**), OFR and corresponding ellipticity versus Rabi frequency of the second control field. $\delta ={\delta }_{na},\,m=1000$ and other parameters are the same with Fig. 2.

To study the effect of control field intensity, we plot the response of the slab versus Rabi frequency of the second control field. Figure 6(a,b) show the intensity of transmission ${T}_{\pm }={|{t}_{\pm }|}^{2}$ and corresponding phase ${\phi }_{\pm }=Arg[{t}_{\pm }]$ versus ${\varOmega }_{c2}$, respectively. As predicted by analytical solutions, for ${\Omega }_{c1}={\Omega }_{c2}$ there is no splitting between two modes and the probe field passes through the slab without OFR. However, different intensities of control fields cause asymmetry in the medium and induce either birefringence or dichroism. The estimation of analytical results (Eqs. (20, 21)) reveals that the minimum values of imaginary parts of susceptibilities are obtained in the detuning

22 $${\delta }^{2}\equiv {\delta }_{na}^{2}=\frac{1}{8}(-{\gamma }^{2}+8\,{\Omega }_{c1}^{2}-8\,{\Omega }_{c1}{\Omega }_{c2}+\sqrt{{\gamma }^{4}-16\,{\gamma }^{2}{\Omega }_{c1}^{2}-64\,{\Omega }_{c1}^{3}{\Omega }_{c2}+16{\Omega }_{c1}{\Omega }_{c2}({\gamma }^{2}+4\,{\Omega }_{c2}^{2})\,}).$$

Assuming such values for detuning of the probe field, the minimum amplitude variations is attained in the slab. So, the birefringence is the main mechanism for rotating the transmitted light. In fact, control fields create an imbalance in susceptibilities of two circularly polarized mods that leads to birefringence and in turn polarization rotation. Consequently, the ellipticity for the selected parameters is very small. Figure 6(b) shows that by increasing the ${\Omega }_{c2}$ to $5\,\gamma$, the phase difference increases and this behavior would be a sign of large OFR. The transmission of linearly polarized modes is also calculated and plotted in Fig. 6(c). Besides, the OFR and corresponding ellipticity are illustrated by Fig. 6(d). Obviously, the probe light is highly transmitted and the polarization of transmitted light rotates by about $\mp {45}^{\circ }$ in $\delta =\pm {\delta }_{na}$. Such a result may be a good achievement for isolator applications. Therefore, intensities of control fields are introduced as a controlling parameter for the optimization of the transmission and OFR.

**Figure 7:**
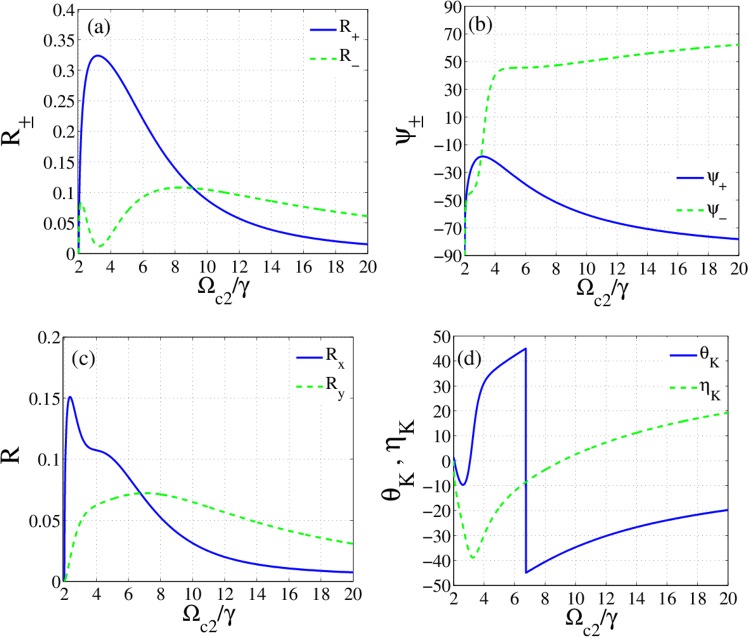
Reflection of circularly polarized modes (**a**), phase profiles (**b**), reflection of linearly polarized modes (**c**) OKE and corresponding ellipticity (**d**) as a function of Rabi frequency of the second control field. Parameters are the same with Fig. 6.

To indicate the role of ${\Omega }_{c2}$ on the reflected beam, Fig. (7) presents reflection (a) and phase (b) of circularly polarized modes, the reflection of linearly polarized (c), OKE and corresponding ellipticity (d). As mention earlier, the asymmetry between the two components of susceptibility depends on the difference between the two laser field intensities. The OKE ${38}^{\circ }$ with zero ellipticity occurs in ${\Omega }_{c2}=9\,\gamma$ where both circular modes are reflected with equal intensity. The greatest conversion into the TM polarization occurs in ${\Omega }_{c2}=7\,\gamma$ where the reflection is about 15% for OKE ${45}^{\circ }$ with very small ellipticity (${6}^{\circ }$). Such results are improvements of the slab response that are obtained by tuning the intensity of the control field.

**Figure 8:**
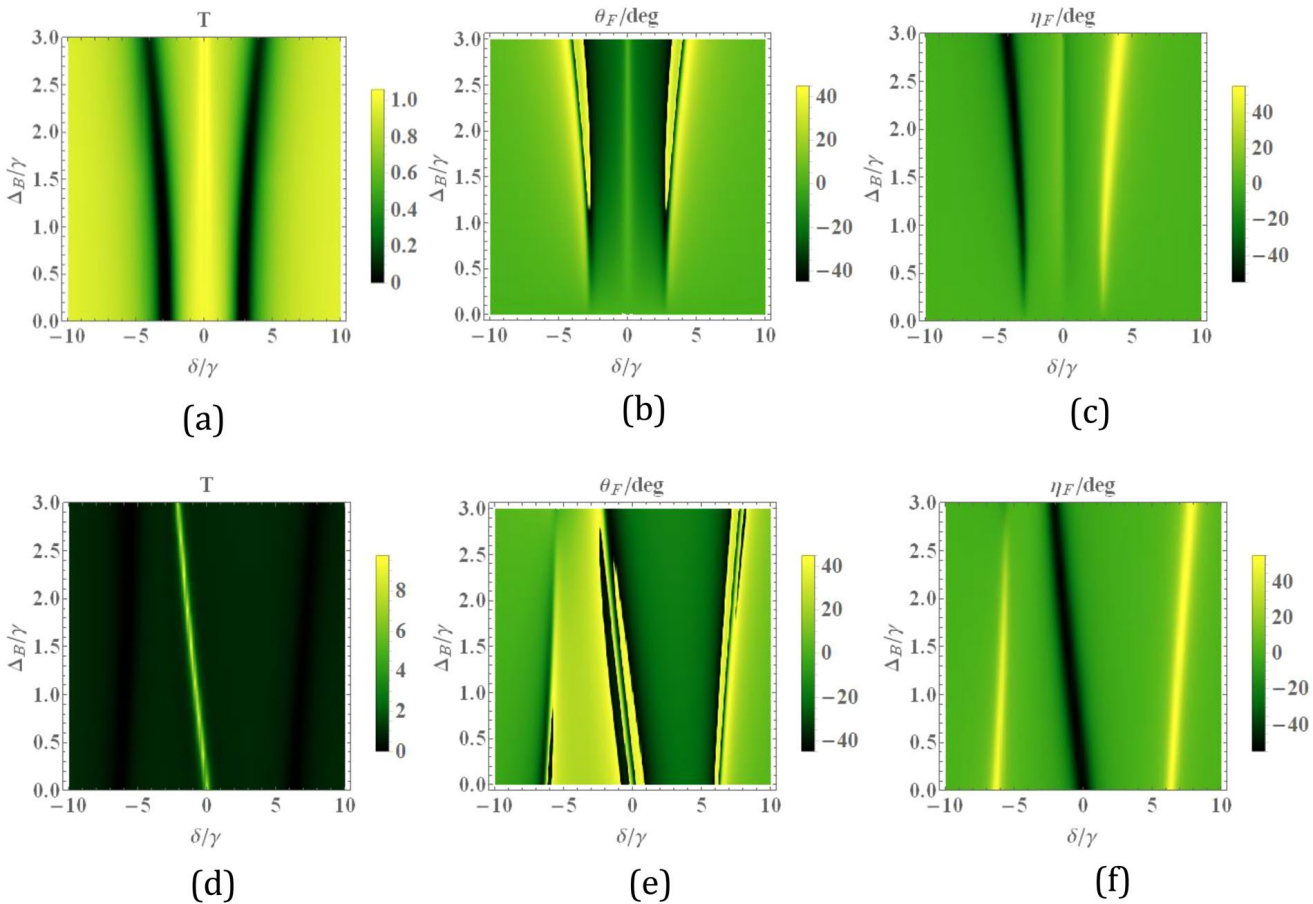
Density plot of the transmission (**a,d**), magneto-optical rotation (**b,e**), and ellipticity (**c,f**) for versus the magnetic field and the detuning of the probe field. The selected parameters are ${\Omega }_{c1}={\Omega }_{c2}=2\gamma$ (first row), and ${\Omega }_{c1}=2\gamma$, ${\Omega }_{c2}=6\gamma$ (second row).The other parameters are the same with Fig. 2.

Finally, we examine the transmission, reflection and corresponding MOR in the presence of a magnetic field. First, we choose equal intensities of the two fields to study the effect of the magnetic field. In this case, the magnetic field lifts the energy degeneracy of the upper states via the Zeeman shift. Figure 8, shows the transmission and MOR as a function of the magnetic field and the probe field detuning. Note that for the equal intensities of control fields and very weak magnetic fields, the Autler-Townes effect occurs for both components of the probe field. So the probe field propagates through the slab without being absorbed in the vicinity of resonance as depicted in Fig. 8(a). For the weak magnetic field, a slight splitting appears between two circular modes. Figure 8(b) shows that by increasing the strength of the magnetic field, MOR increases. In ${\varDelta }_{B}\cong 3\,\gamma$ and $\delta \cong 1.5\,\gamma$, the transmission 80% is achieved for MOR $-{25}^{\circ }$ with no ellipticity (Fig. 8(c)). Now, we choose the different intensities of control fields and plot the total transmission (d), MOR (e), and ellipticity (f) versus the magnetic field and the detuning of the probe field. This setting of external fields provides two different mechanisms for the generation of asymmetry. It is to be expected that the influences of control laser and magnetic fields modify the response of the slab. The magnetic field causes a minimum shift of the transmission curve toward the negative detunings of the probe field. Also, Fig. 8 (d,e) represent the high transmission (100%) of the probe field with large MOR (${45}^{\circ }$) and very small ellipticity at ${\Delta }_{B}\cong 1\,\gamma$ and $\delta \cong -5\,\gamma$.

**Figure 9:**
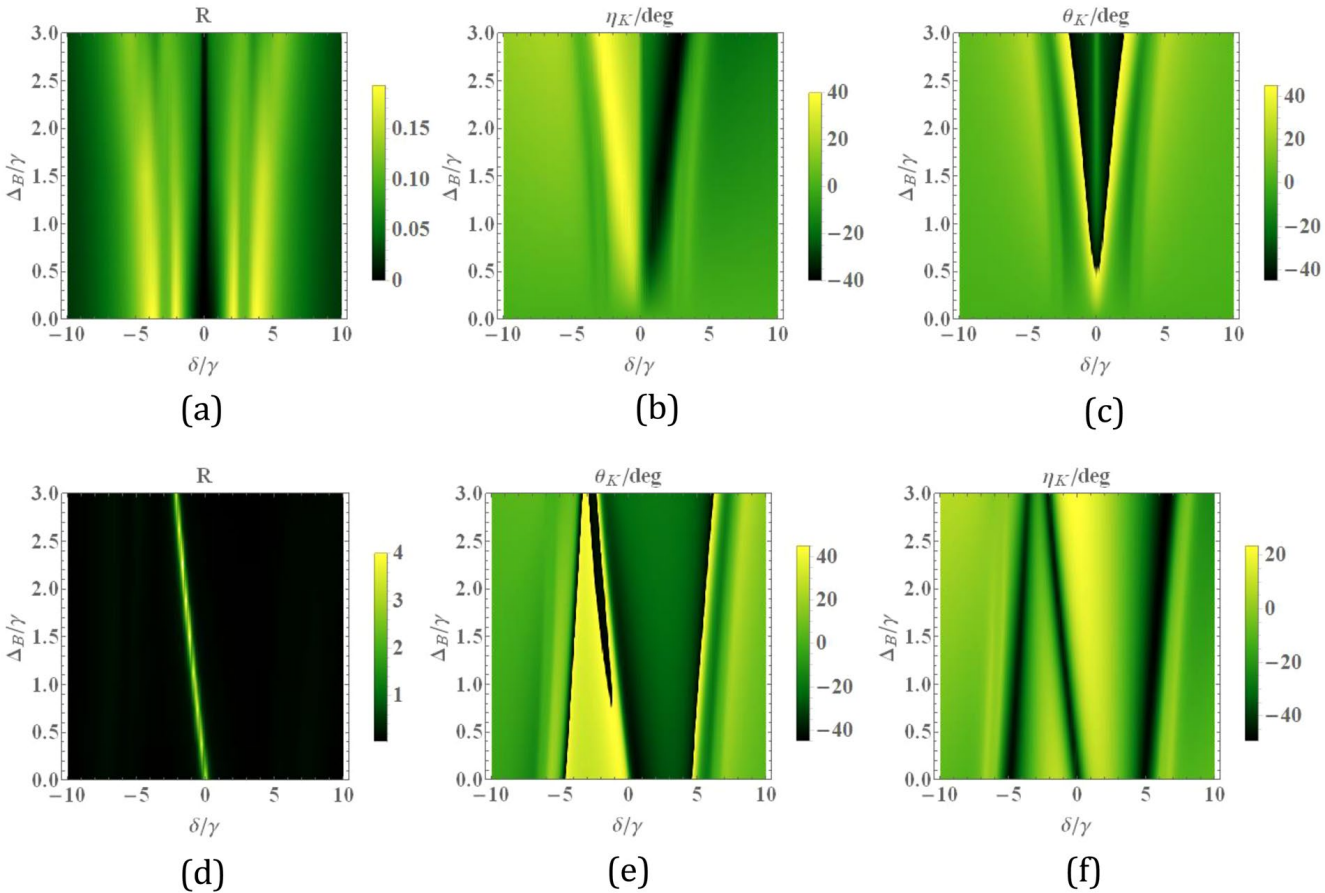
Density plot of the reflection (**a,b**), magneto-optical Kerr rotation (**c, d**) and ellipticity versus the magnetic field and the detuning of the probe field. The selected parameters are ${\Omega }_{c1}={\Omega }_{c2}=2\gamma$ (first row), and ${\Omega }_{c1}=2\gamma$, ${\Omega }_{c2}=6\gamma$ (second row).The other parameters are the same with Fig. 2.

In order to study the effect of the magnetic field on the reflected beam, we present the total reflection (a, d), MOKE (b, e), and ellipticity (c, f) versus the magnetic field and the detuning of the probe field in Fig.  9. The first (second) row shows the results for the equal (different) intensities of the control fields. It is seen that large MOKE with very small reflection is obtained for equal intensities of control fields. Including asymmetry induced by the control laser fields (second row), we succeed in obtaining an especially desirable result that is MOKE ${45}^{\circ }$ for close to 100% reflection around ${\Delta }_{B}=1.5\gamma ,\,\delta \cong 1.5\gamma$. Note that he total transmission larger than unity and high reflection arise for the probe field due to the amplification of light beam via doped atoms.

## Conclusions

In this paper, we investigate the rotation of polarization plane of light that passes through a slab doped with double-V type atoms. In the absence of the magnetic field, OFR and OKE occur for light due to the control laser field induced asymmetry in the atomic system. We first study the influence of a circularly polarized control field on the atomic response and show that one of the probe field modes propagates in the system without being absorbed. In such a case, asymmetry is achieved as well as transparency for one of the probe field components. By switching on the second control field, the coherences like cross-Kerr effect appear in the slab that leads to enhancement of OFR and OKE. One of the significant observed properties is the amplification of light via atoms, resulting in the transmission larger than unity. Using this feature, OFR and OKE can be obtained with higher output intensity of light. In the presence of an external magnetic field, the rotation of the polarization plan enhances. MOR ${45}^{\circ }$ with transmission close to $100 \%$ and large MOKE with high reflection from a small device makes the proposed model as a great candidate for use as a simultaneous Faraday and Kerr rotator.
